# Case Report: Co-existence of sarcoidosis and Takayasu arteritis

**DOI:** 10.12688/wellcomeopenres.15837.2

**Published:** 2020-07-23

**Authors:** Jiwan Poudel, Ujjwol Risal, Keshav Raj Sigdel, Buddhi Prasad Paudyal, Sudeep Adhikari, Buddha Basnyat

**Affiliations:** 1 Internal Medicine, Patan Academy of Health Sciences, Lalitpur, Nepal; 2 Oxford University Clinical Research Unit, Patan Academy of Health Sciences, Lalitpur, Nepal

**Keywords:** Sarcoidosis, takayasu arteritis, coexistence

## Abstract

Takayasu arteritis is a rare systemic large vessel vasculitis affecting the aorta and its branches. Sarcoidosis, too, is an inflammatory disease. Both entities are granulomatous conditions with a questionable association in their etiopathogenesis. Only a few cases of their coexistence have been reported in the literature. To our knowledge, no such cases have been reported from Nepal. We report a Nepalese woman who presented with non-productive cough, progressive shortness of breath and chest tightness of 3 years duration. She had a history of recurrent bilateral granulomatous uveitis over the previous 3 years. Examination revealed clubbing of digits, absent pulses over the left radial, ulnar and brachial arteries, and a weak pulse over the right arm including the bilateral carotid arteries. Pulmonary function test showed restrictive pattern, a high-resolution computed tomography (HRCT) scan of the chest revealed findings suggestive of pulmonary sarcoidosis. A CT angiogram suggested large vessel vasculitis. Bronchoscopy with biopsy revealed granulomatous inflammation, negative for malignancy and tuberculosis. She was hence, diagnosed with co-existing Takayasu arteritis and sarcoidosis, and treated with Prednisolone 60 mg once daily with dramatic improvement over 4 days and was discharged stable on domiciliary oxygen. She is currently on azathioprine 50 mg, prednisolone 10 mg without the need for supplemental oxygen. This case report highlights the importance of a proper physical examination as a guide to the use of modern technology in making a correct diagnosis. Furthermore, in countries where tuberculosis is endemic, it should always come as the most important differential diagnosis of granulomatous inflammation.

## Background

Sarcoidosis is a systemic inflammatory disorder of unknown etiology characterized by non-caseating, granulomatous inflammation ^1^. It affects young adults 20 to 40 years of age ^2^. Takayasu arteritis (TA) is a vasculitis involving large sized arteries, mainly the aorta and its branches. It is also an inflammatory disease of unknown etiology characterized by granulomatous vasculitis ^3^. Sarcoidosis and TA are unrelated diseases; however rare coexistence has been noted ^4– 11^. It is not clear if the coexistence is due to chance alone or if there is some common pathogenetic mechanism related to both the diseases. We report a patient from Nepal who was diagnosed with sarcoidosis and TA concomitantly, and aim to contribute to literature regarding patients with these coexisting diseases.

## Case Presentation

A 40-year-old Asian woman, non-smoker, housewife, presented in December, 2018 with progressive shortness of breath, initially only during physical exertion (i.e, walking upstairs) which later started occurring even at rest and a non-productive cough of three years duration which worsened in the ten days preceding presentation. She also complained of chest tightness during this presentation. She did not have a history suggestive of orthopnea or paroxysmal nocturnal dyspnea, swelling of her legs and/or body, fever, blood mixed sputum, chest pain or joints pain. She did not have exposure to patients with pulmonary tuberculosis. She had presented to an ophthalmologist 3 years back due to pain and redness of both eyes and was later diagnosed with bilateral granulomatous uveitis. She was treated with topical steroid for the same duration with intermittent intervals of as long as 2–3 months.

On examination, she was afebrile with a blood pressure of 130/80 mmHg in the right arm and heart rate was 80/minute. She had clubbing of her digits. Examination of peripheral pulses revealed absent pulse over the left radial and brachial arteries and feeble pulse over the right radial and brachial arteries, and bilateral carotid arteries. Carotid bruit was heard on the left side. Her oxygen saturation at room air was only 76% and required 4 liters/minute of oxygen to maintain 92% saturation (Normal >95%). Her jugular venous pressure was not raised and there was no pedal or sacral edema.

Laboratory parameters with normal ranges in parenthesis, are as follows:

Complete blood count: white cell count 4.2 (4–10) × 10^9/L; neutrophils 78%; lymphocytes 20%; red blood cells 4.6 (4.2–5.4) × 10^12/L; haemoglobin 13.3 (12–15) g/dL; platelets 244 (150–400) × 10^9/L.

Biochemistry: random blood sugar 126 (65–110) mg/dL; urea 29 (17–45) mg/dL; creatinine 0.9 (0.8–1.3) mg/dL; sodium 139 (135–145) mmol/L and potassium 4.3 (3.5–5) mmol/L.

Hepatic panel: bilirubin total 0.8 (0.1–1.2) mg/dL and direct 0.5 (0–0.4) mg/dL; alanine transaminase 39 (5–30) units/L; aspartate transaminase 45 (5–30) units/L; alkaline phosphatase 103 (50–100) IU/L; albumin 2.6 (3.5–5) g/dL

Erythrocyte sedimentation rate (ESR) was elevated at 45 mm/hr and c-reactive protein (CRP) was 4 mg/L (Normal < 10 mg/L). Angiotensin converting enzyme (ACE) levels were elevated at 186 u/L (Normal < 52 u/L); rheumatoid factor and anti-nuclear antibodies by indirect immunofluorescence were negative. Sputum examination did not reveal any organisms including acid fast bacilli. Sputum analysed by gene Xpert also did not detect *Mycobacterium tuberculosis*. The electrocardiogram was normal. Echocardiography showed moderate tricuspid regurgitation with moderate pulmonary arterial hypertension with a normal systolic function.

**Figure 1.**
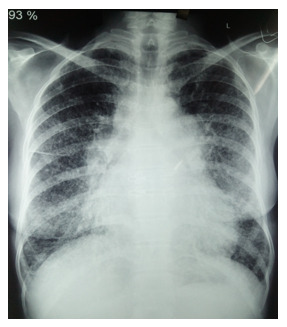
Chest X-ray showing bilateral reticulo-nodular infiltrates involving the middle and lower zones and some parts of the upper zones.

**Figure 2.**
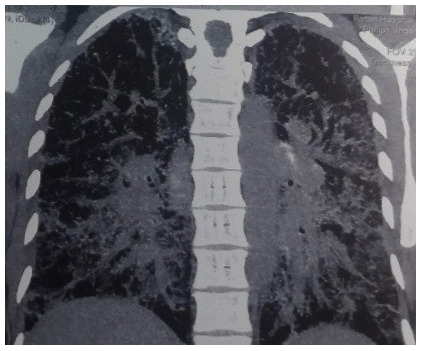
High-resolution computed tomography (HRCT) chest showing diffuse ground glass changes with interlobular septal thickening with mosaic attenuation and multiple enlarged calcified mediastinal and hilar lymph nodes.

**Figure 3.**
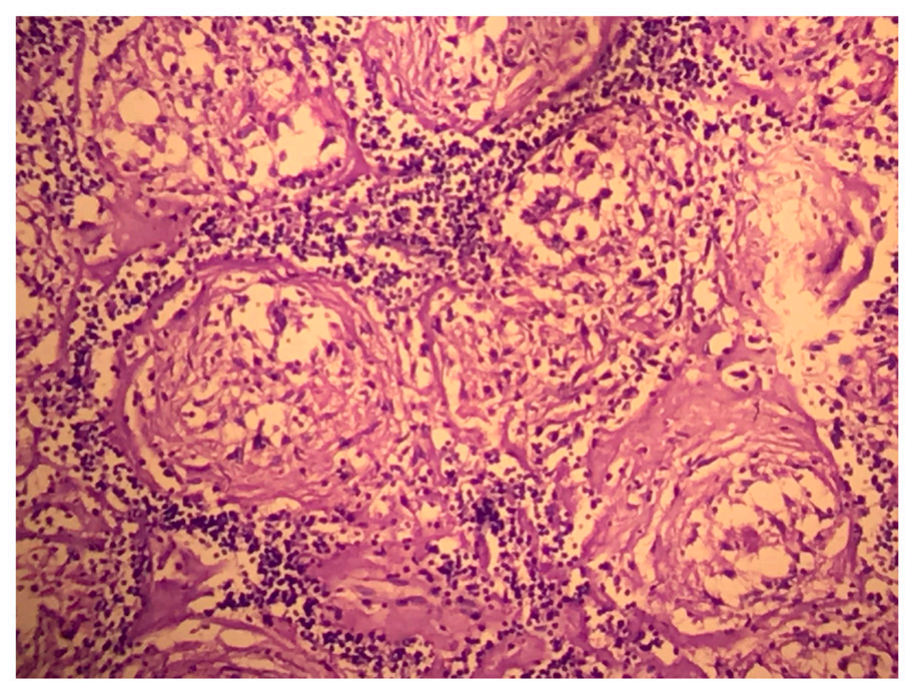
Lung biopsy showing large non- caseating granulomas with tightly packed central area composed of epithelioid cells, multinucleated giant cells and T- lymphocytes.

**Figure 4.**
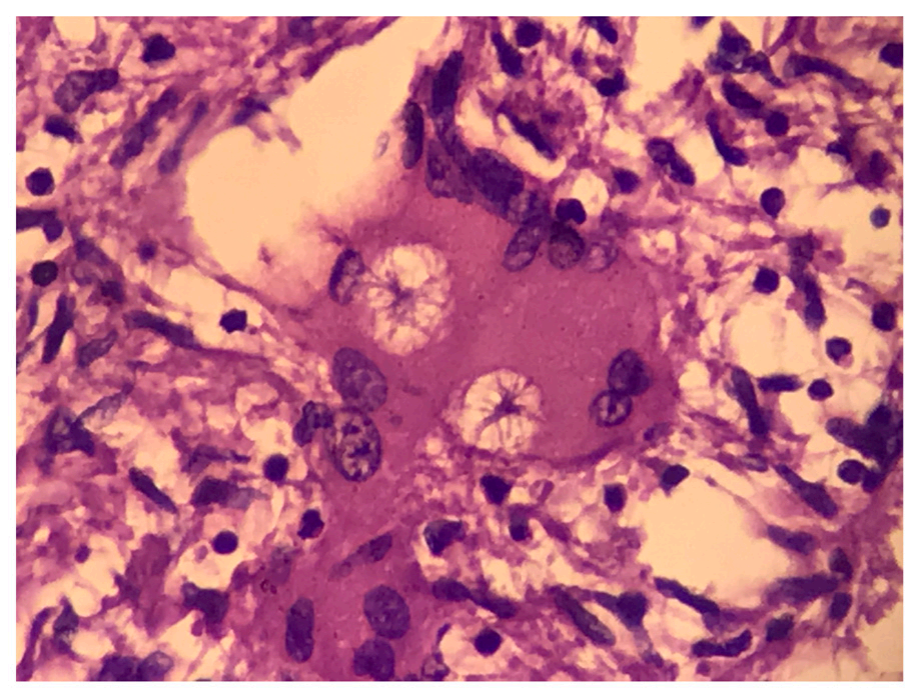
Further magnification of the granuloma revealing the characteristic ‘asteroid bodies’.

Chest X-ray ( Figure 1) revealed bilateral reticulo-nodular infiltrates involving the middle and lower zones and some parts of the upper zones. No changes were noted compared to a radiograph done three years ago. The pulmonary function test showed a restrictive pattern. High resolution computed tomography (CT) of the chest ( Figure 2) showed diffuse ground glass changes with interlobular septal thickening with mosaic attenuation and multiple enlarged calcified mediastinal and hilar lymph nodes (separate image of mediastinal section could not be retrieved). Bronchoscopy with trans-bronchial lung biopsy revealed non- caseating granulomatous inflammation with characteristic ‘asteroid bodies’ ( Figure 3 and Figure 4), and was negative for malignancy and tuberculosis. Hence the diagnosis of sarcoidosis (pulmonary and ocular) was made.

**Figure 5.**
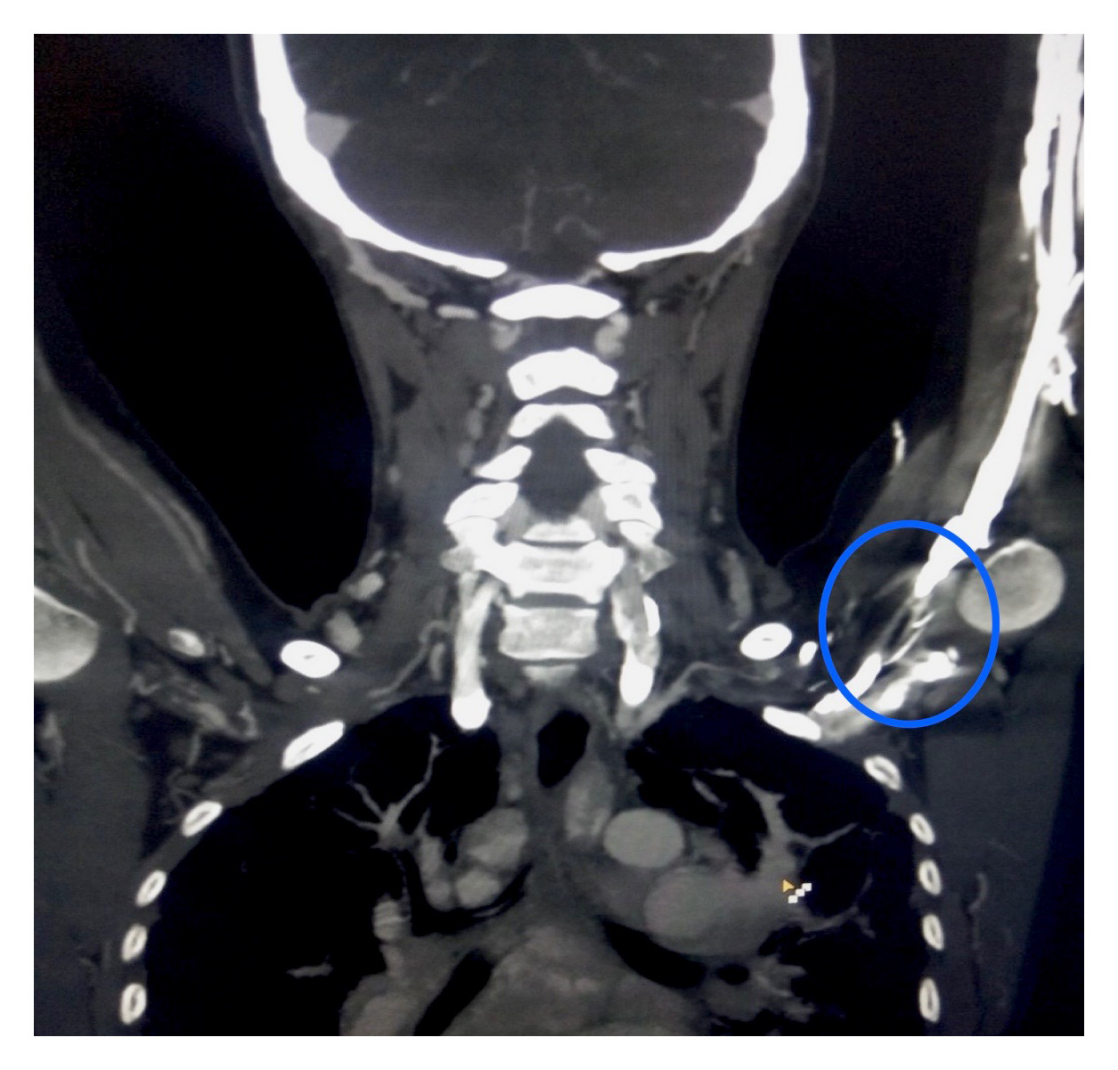
Computed tomography (CT) aortogram showing stenosis of left subclavian artery with almost complete block at distal part and proximal left axillary artery.

The findings of abnormal pulse led us to do a CT angiogram of aorta and its branches. Figure 5 shows stenosis of the left subclavian artery with almost complete block at the distal part as well as the proximal left axillary artery. Though images not available for the report, there was stenosis of right axillary, brachial artery and its branches, with sparing of abdominal aorta up to the lower extremities. This was suggestive of large vessel vasculitis in the upper extremities, Takayasu arteritis (Type I).

With the diagnosis of sarcoidosis with coexisting Takayasu arteritis, she was treated with prednisolone 60 mg once daily (1 mg/kg/day) along with supportive treatment including oxygen, chest physiotherapy, bone protection with calcium and vitamin D, and thromboembolism prophylaxis with daily 60 mg of subcutaneous enoxaparin. Ophthalmology examination showed bilateral granulomatous uveitis and was treated with topical prednisolone drops. She dramatically improved over 3–4 days and required only 1 liter/minute of oxygen support. Pneumococcal vaccination was administered. She was later discharged on domiciliary oxygen with a tapering prednisolone dosage.

After four weeks of discharge, while she was still on 30 mg of prednisolone, she developed blurring of vision. Ophthalmologic examination showed increased intraocular pressure with findings suggestive of Secondary glaucoma, probably steroid induced. Topical steroid was stopped and since oral steroid was to be tapered off quickly, azathioprine 50 mg/day was started as a steroid sparing immunosuppressant. Glaucoma was treated with topical timolol (0.25% twice daily) and oral acetazolamide (500 mg followed by 250 mg twice daily), and she responded well over the course of 2 weeks.

The patient is still on regular follow up (after 15 months of diagnosis) and is currently asymptomatic. She does not require oxygen and does not have symptoms of vasculitis (carotidynia, headache, lightheadedness, vertigo, tingling, numbness, limb claudication, cyanosis, arthralgias and skin lesions like erythema nodosum). She is currently taking azathioprine 50 mg/day, prednisolone 10 mg/day with calcium and vitamin D supplements. She is also on regular follow up every 3 months with a pulmonologist.

## Discussion

Our patient was symptomatic with dyspnoea and cough for the 3 years prior to presentation but was not evaluated. The development of pain and redness in both eyes led her to visit an ophthalmologist who diagnosed bilateral granulomatous uveitis 3 years previous. Sarcoidosis was unfortunately missed during the initial presentation, allowing the patient to receive topical steroid therapy alone for 3 years which allowed the systemic disease to flourish. Lungs are most commonly (95%) involved in sarcoidosis ^12^, and almost 30% have extrapulmonary manifestations ^13^ with ocular involvement in 25% of patients. Almost 5% of patients have ocular involvement on presentation. Granulomatous uveitis is the intraocular manifestation of sarcoidosis. Finally, the respiratory symptoms in the background of granulomatous uveitis led us to suspect sarcoidosis and the chest imaging (radiographic stage II-III on chest x-ray) and bronchoscopic examinations helped confirm the diagnosis. Systemic glucocorticoid treatment was started due to severe involvement of the lungs. Due to the unavailability of bronchoscopy in our center, we had to refer the patient to a different center which delayed the diagnosis by a few days.

Secondary glaucoma is a common complication in intraocular sarcoidosis as seen in our patient, which occurred due to prolonged use of topical steroid eye drops. In our patient, we stopped prednisolone drop, treated glaucoma with timolol and acetazolamide, tapered off the systemic prednisolone quickly and started her on azathioprine as a steroid-sparing agent. The initial management of sarcoidosis involves the use of systemic glucocorticoid for inducing remission. The use of immunosuppressive agents such as methotrexate ^14^, azathioprine ^15^ and leflunomide ^16^ is reserved for cases where there is a lack of response or side effects to glucocorticoids ^17^.

Our patient also had coexisting Takayasu arteritis which is a large vessel granulomatous vasculitis. The incidental finding of weak pulses and carotid bruits during the physical examination in a young Asian woman (in whom atherosclerotic vascular disease would be uncommon) led to the suspicion of large vessel vasculitis. CT angiography later helped to make the proper diagnosis. The diagnosis of Takayasu arteritis can be effectively made based on typical clinical findings (discrepant blood pressure, absent or feeble peripheral pulses, arterial bruits) and imaging findings of narrowing of the aorta and/or its primary branches, without the need for demonstration of granulomatous vasculitis in histopathology ^18^. The overall treatment approach remains similar to sarcoidosis (using systemic steroid and alternative steroid sparing immunosuppressants as required).

As Nepal is an endemic region for tuberculosis, and the fact that tuberculosis can mimic findings of sarcoidosis and also of large vessel vasculitis, tuberculosis was a strong all-encompassing diagnosis that could have explained the abnormal findings in our patient. But tuberculosis was effectively ruled out with sputum and after bronchoscopic examination. Our patient presented with respiratory symptoms which led to the diagnosis of sarcoidosis, but she also had other incidental findings not in keeping with sarcoidosis alone. Hence we entertained the possibility of another disease. Takayasu-like vasculitis has been reported to be associated with sarcoidosis, but direct association have not been established.

The coexistence of Takayasu arteritis with sarcoidosis is rare, and has been reported in a few case reports ^4– 11^. Though these two diseases are unrelated, there is a new hypothesis that some unifying immunological mechanism may be responsible for the concomitant presence of the two diseases ^8^. Until now, both are considered to be granulomatous inflammatory conditions of unknown etiology. However, some studies have pointed various possible common immune mechanism for these two entities, including mycobacterial heat shock proteins (HSPs). These mycobacterial HSPs are thought to cross react with human HSPs due to their molecular mimicry, thereby producing an increased abnormal immune response. A meta-analysis by *Fang C. et al*. ^19^ hypothesized that some mycobacterial antigens contribute to immune-mediated granulomatous inflammation (sarcoidosis). In a study by *Aggarwal et al.* ^20^ including 36 Takayasu arteritis patients, they found enhanced humoral immune response to mycobacterial antigens compared to healthy controls. Similarly, *Kumar Chauhan et al*. ^21^ in a study of 26 patients with Takayasu's disease, found a greater proliferation of CD _4_ T-lymphocytes in the presence of mycobacterial HSP-65 compared to controls, as well as higher levels of IgG antibodies directed against the anti-HSP-65 and anti-HSP-60. Put together, microbial involvement seems to play an important role in granulomatous inflammation. But this needs further evaluation with a larger cohort of patients with coexisting sarcoidosis and Takayasu arteritis.

## Conclusion

Sarcoidosis should be suspected in young adults who present with progressive respiratory and ocular symptoms. But large vessel vasculitis causing abnormal pulse and arterial bruits, is distinctly rare in sarcoidosis. The incidental findings of weak pulses and carotid bruits in this young woman therefore led us to the diagnosis of coexisting Takayasu arteritis. This case report highlights the importance of a proper physical examination, even as a guide to modern technology to make the correct diagnosis. The coexistence of sarcoidosis and Takayasu arteritis is rare, and needs further case report compilations to help delineate this association, if any. Importantly, in our setting, tuberculosis, the great mimicker, is rampant and always has to be ruled out in a patient with clinical features like ours.

## Consent

Written informed consent for publication of clinical details and clinical images was obtained from the patient.

## Data availability

### Underlying data

All data underlying the results are available as part of the article and no additional source data are required.
