# Supplementary material for: Case Report: Co-existence of sarcoidosis and Takayasu arteritis
Source: Wellcome Open Res. 2020 Jul 23;5:73. Originally published 2020 Apr 17. [Version 2] doi: 10.12688/wellcomeopenres.15837.2 (PMC7450474; doi:10.12688/wellcomeopenres.15837.2)
Supplement: Peer review history

# Peer review: Reviewer response for version 2

**DOI:** 10.21956/wellcomeopenres.17770.r40058

**Published:** 2020-08-26

**Authors:** Hollis E. Krug

**Affiliations:** 1 Rheumatology Department, Veterans Affairs Medical Center, Minneapolis, MN, USA; 2 University of Minnesota Medical School, Minneapolis, MN, USA

This is a well-written report of a rare case of co-existent Takayasu’s Arteritis and sarcoidosis. It would benefit from an improved Figure 2 as the one included is of poor quality. The previous diagnosis of granulomatous uveitis could have more discussion including other diseases that could affect the pulmonary and vascular system such as GPA. There is no mention of ANCA testing. The dramatic response to steroids alone supports the diagnoses of both TA and sarcoidosis. The discussion regarding the necessity of ruling out TB is important and well done as is the summary of pathophysiology of both TA and sarcoidosis. I agree with the editorial changes already suggested.

Are enough details provided of any physical examination and diagnostic tests, treatment given and outcomes?

Yes

Is the case presented with sufficient detail to be useful for other practitioners?

Yes

Is sufficient discussion included of the importance of the findings and their relevance to future understanding of disease processes, diagnosis or treatment?

Yes

Is the background of the case’s history and progression described in sufficient detail?

Yes

Reviewer Expertise:

Gout, ankylosing spondylitis, osteoarthritis, pain.

I confirm that I have read this submission and believe that I have an appropriate level of expertise to confirm that it is of an acceptable scientific standard.

# Peer review: Reviewer response for version 1

**DOI:** 10.21956/wellcomeopenres.17372.r38502

**Published:** 2020-06-15

**Authors:** Debashish Danda

**Affiliations:** 1 Department of Clinical Immunology and Rheumatology, Christian Medical College, Vellore, Vellore, Tamil Nadu, India

- Acceptable now. Clinical presentation and discussion is relevant. It is a rare association of a rare disease. Tuberculosis as differential has been discussed adequately.
- Images are provided for the readers.
- Although not an exceptional case, it may be still useful for primary and secondary care physicians. Both sarcoidosis and Takayasu are granulomatous inflammatory diseases, hence it is interesting.

Are enough details provided of any physical examination and diagnostic tests, treatment given and outcomes?

Yes

Is the case presented with sufficient detail to be useful for other practitioners?

Yes

Is sufficient discussion included of the importance of the findings and their relevance to future understanding of disease processes, diagnosis or treatment?

Yes

Is the background of the case’s history and progression described in sufficient detail?

Yes

Reviewer Expertise:

Takaysu arteritis, Sjogren syndrome, Lupus & spondyloarthropathies

I confirm that I have read this submission and believe that I have an appropriate level of expertise to confirm that it is of an acceptable scientific standard.

# Response

**Published:** 2020-07-21

**Authors:** Sudeep Adhikari

**Affiliations:** Patan Academy of Health Sciences, Nepal

Thank you sir for the review.

# Peer review: Reviewer response for version 1

**DOI:** 10.21956/wellcomeopenres.17372.r38723

**Published:** 2020-06-03

**Authors:** Varun Dhir

**Affiliations:** 1 Department of Internal Medicine, Postgraduate Institute of Medical Education and Research, Chandigarh, Punjab and Haryana, India

This is a well written and interesting case report about the coexistence of sarcoidosis and takayasu arteritis. The images are excellent.

 However, I have the following suggestions:

1. The current image 5, angiogram, the authors could get a reconstructed 3D image instead of this single coronal plane. Also in the current image, the right subclavian and the carotids are also not visualized.
2. Figure 2 is again not very good and may suggest giving separate mediastinal sections and lung sections - axial.
3. There are some minor language suggestions:
  - rather than "The coexistence of Takayasu arteritis with sarcoidosis is rare, and has been ***presented*** in a few case reports", it could be better as ***reported.***
  - vessel vasculitis causing abnormal pulse and arterial bruits, is ***not common*** in sarcoidosis." replaced by ***distinctly rare.***

Are enough details provided of any physical examination and diagnostic tests, treatment given and outcomes?

Yes

Is the case presented with sufficient detail to be useful for other practitioners?

Yes

Is sufficient discussion included of the importance of the findings and their relevance to future understanding of disease processes, diagnosis or treatment?

Yes

Is the background of the case’s history and progression described in sufficient detail?

Yes

Reviewer Expertise:

Rheumatology and Clinical Immunology

I confirm that I have read this submission and believe that I have an appropriate level of expertise to confirm that it is of an acceptable scientific standard, however I have significant reservations, as outlined above.

# Response

**Published:** 2020-07-21

**Authors:** Sudeep Adhikari

**Affiliations:** Patan Academy of Health Sciences, Nepal

Thank you sir for the review.

 We have made changes in the manuscript as per your suggestions.

 However, the images showing most of the abnormalities clearly could not be retrieved for reporting due to technical issues, and we have acknowledged that in the revised manuscript.
